# DFFNDDS: prediction of synergistic drug combinations with dual feature fusion networks

**DOI:** 10.1186/s13321-023-00690-3

**Published:** 2023-03-16

**Authors:** Mengdie Xu, Xinwei Zhao, Jingyu Wang, Wei Feng, Naifeng Wen, Chunyu Wang, Junjie Wang, Yun Liu, Lingling Zhao

**Affiliations:** 1 Department of Medical Informatics, School of Biomedical Engineering and Informatics, Nanjing Medical University, Nanjing, China; 2 Institute of Medical Informatics and Management, Nanjing Medical University, No. 300 Guang Zhou Road, Nanjing, 210029 China; 3 Department of Information, First Affiliated Hospital of Nanjing Medical University, Nanjing, 210029 China; 4 Faculty of Computing, Harbin Institute of Technology, Harbin, China; 5 School of Mechanical and Electrical Engineering, Dalian Minzu University, Dalian, China; 6 Department of Epidemiology, School of Public Health, Nanjing Medical University, Nanjing, China

**Keywords:** Drug combination, Synergistic effect, Deep learning, Dual-feature fusion

## Abstract

Drug combination therapies are promising clinical treatments for curing patients. However, efficiently identifying valid drug combinations remains challenging because the number of available drugs has increased rapidly. In this study, we proposed a deep learning model called the Dual Feature Fusion Network for Drug–Drug Synergy prediction (DFFNDDS) that utilizes a fine-tuned pretrained language model and dual feature fusion mechanism to predict synergistic drug combinations. The dual feature fusion mechanism fuses the drug features and cell line features at the bit-wise level and the vector-wise level. We demonstrated that DFFNDDS outperforms competitive methods and can serve as a reliable tool for identifying synergistic drug combinations.

**Supplementary Information:**

The online version contains supplementary material available at 10.1186/s13321-023-00690-3.

## Introduction

Drug therapy is the most commonly used method in clinical cancer treatments. To address clinical demands, the number of anticancer drugs has increased rapidly, and many efficient single drugs have been applied in cancer therapy. Although monotherapy has contributed greatly to developing disease treatments, it has some drawbacks due to the heterogeneity of drug responses, such as toxicity and drug resistance [1]. Drug combinations, which involves using two or more drugs to treat a specific disease, have been proposed as valid treatment approaches [2]. Combination methods allow different drugs to target various targets and pathways, thereby improving the treatment effects, reducing side effects and decreasing drug resistance [3, 4]. Therefore, drug combinations have been suggested as a potential strategy for addressing drawbacks such as heterogeneity.

Various methods for identifying valid drug combinations have been proposed. The traditional testing method involves clinical trials; however, only a small number of drugs are investigated through clinical trials, as they are time-consuming, expensive, and might expose patients to unnecessary treatment [5]. Therefore, the high-throughput drug screening method [6] has been applied to screen effective drug combinations. High-throughput drug screening method allows automated testing of chemical and biological compounds for specific biological targets and accelerates the identification of synergistic drug combinations. However, high-throughput drug screening methods have failed to reveal the action modes of drug molecules in vivo [7], it is impractical to screen all possible drug combinations for all possible indications. Therefore, several computational methods have been proposed to address the significant increase in the number of available drugs. These computational methods include systems biology methods [8], kinetic models [9] and machine learning methods [10]. Among them, machine learning methods have powerful modeling capabilities because machine learning approaches can learn potential drug features, allowing these models to effectively predict the synergistic effects of various drug combinations while reducing the costs of drug trials. Thus, machine learning has developed rapidly in this field.

Machine learning methods can be divided into two categories: classical machine learning and deep learning. The most commonly used classical machine learning methods are random forests [11], extreme gradient boosting [12] and support vector machines [13]. Li proposed a random forest-based drug combination synergy prediction model on the basis of drug-target networks and drug-induced gene expression profiles to predict synergistic anticancer combinations [14]. Sidorov et al. [15] proposed an XGBoost-based model. However, this approach trains a unique model for each cell line rather than a single model for all cell lines; thus, differences in the cell lines may reduce the root-mean-square error by up to 50%, thereby decreasing the reliability of the model. Julkunen et al. [16] proposed comboFM, a drug combination prediction model that models cell context-specific drug interactions through higher-order tensors and efficiently uses factorization machines to learn tensor latent factors. This model can predict the responses of new drug combinations and explore different drug combination doses.

While classical machine learning relies on handcrafted features, deep learning approaches can extract features from raw data without handcrafted feature extraction. Various neural networks have been proposed, including the convolutional neural network (CNN) [17], recurrent neural network (RNN) [18], and attention mechanism [19]. These neural networks have been successfully applied in computer vision [20] and natural language processing (NLP) tasks [21]. Furthermore, deep learning has gradually been applied in the field of drug prediction. Preuer et al. [5] utilized DeepSynergy, a deep learning method based on a feedforward network. This model uses molecular fingerprints and cell line gene expression. This approach was the first attempt to utilize deep learning in this domain, and this model achieved better performance than traditional machine learning methods. Yang et al. [22] proposed GraphSynergy, a new model for identifying synergistic combinations. This model adapts a spatial-based graph convolutional network to encode higher-order structural information of protein modules targeted by drug pairs and the protein modules associated with specific cancer cell lines in protein-protein interaction (PPI) networks. Jiang et al. [23] proposed using a graph convolutional network to predict drug combinations in cancer cell lines. In 2021, the DeepDDS model [24] was proposed, which uses a graph neural network and attention mechanism to identify valid drug combinations. In this model, to obtain cell structures, RDKit is applied to convert simplified molecular input line entry specifications (SMILES) into molecular graphs, and the structures and gene expression patterns are integrated to identify synergistic combinations. However, some problems remain. In terms of feature extraction, these methods do not sufficiently investigate the SMILES information. Moreover, in terms of feature fusion, the abovementioned methods simply concatenate drug features and cell line features, and these fusion methods do not fully capture the interactions between these features.

Therefore, in this paper, we proposed the dual feature fusion network for drug-drug synergy prediction (DFFNDDS), a deep learning model for predicting the synergistic effects of drug combinations. The model inputs are the SMILES representations of the drugs, hashed atom pair fingerprints of the drugs, and cell line gene expression. The model output is the synergy score of the given drug combination. To address the above problems, we investigated the SMILES representations and used a fine-tuned BERT model to identify efficient drug features. To obtain the fusion features, we used a double-view feature fusion mechanism to combine the drug and cell line features. Finally, we compared our method to recent deep learning prediction models, including MatchMaker [25], DeepSynergy [5], EPGCNDS [26], GCNBMP [27] and DeepDDS [24], on the benchmark datasets DrugComb [28] and DrugCombDB [29]. The experimental results indicated that DFFNDDS is an effective model for predicting the synergistic scores of drug combinations.

## Methods and pipelines

### Pipeline

**Fig. 1:**
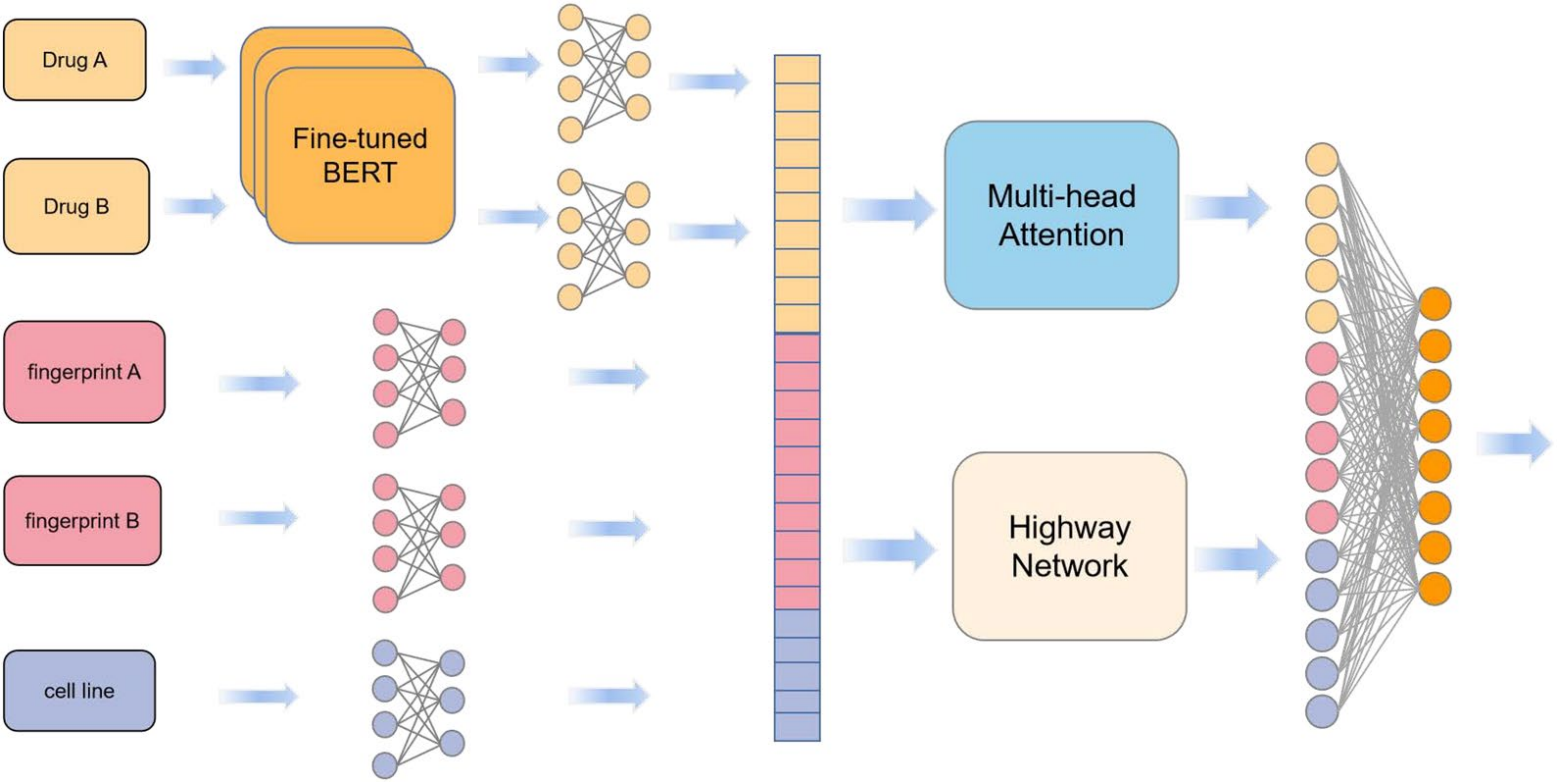
The architecture of DFFNDDS

Figure 1 illustrates the end-to-end learning framework for predicting drug combinations. Our framework has 4 modules, including the SMILES encoder, dimensional alignment module, dual fusion module, and predictor module. For each pairwise drug combination, the input layer receives the SMILES string representations, hashed atom pair fingerprints of the two drugs, and cancer cell lines addressed by the drugs. Then, the SMILES string is encoded by a fine-tuned BERT model that converts the features into vectors. Moreover, the gene expression in the cell lines, output of the SMILES encoder and hashed atom pair fingerprints are input into the dimensional alignment module, which maps the inputs to the same dimension. To fuse the features, we utilize two networks (multi-head attention mechanism and highway network) to extract and combine the input features in the dual fusion block. Finally, the outputs of the two networks are concatenated to obtain the final feature representation, which is propagated through the linear layer. The output of the linear layer is the predicted synergy score, which is used to determine whether the drug combination is synergistic or antagonistic.

### Drug encoding based on SimCSE

In recent years, pretrained models have thoroughly changed various artificial intelligence domains, including NLP. BERT (bidirectional encoder representations from transformers) [30] is one of the most famous NLP models. BERT includes 12 transformer encoders and uses a masked language model to predict randomly masked words in a sequence. BERT can learn both left and right context with the addition of an attention mechanism. Moreover, BERT has achieved state-of-the-art performance on eleven NLP tasks. Inspired by the great NLP performance, many chemical language models have been proposed in the field of drug discovery to predict drug molecule characteristics and protein-protein interactions (PPIs). For instance, ChemBERT [31], DeepChem [32], and SciBERT [33] were developed to apply deep learning in drug discovery. BERT models use 3 common methods to generate the embedding of the input sentence: cls pooling, max pooling and mean pooling. These three methods cannot completely extract textual information [34]. Thus, to enhance the encoding quality, we use simple contrastive learning of sentence embeddings (SimCSE) [35] to fine-tune the original BERT model. The SimCSE framework uses contrastive learning objectives to fine-tune the BERT model and has achieved competitive results on NLP tasks. This fine-tuned model takes SMILES to predict itself in a contrastive objective, using only standard dropout as noise, we apply this method to generate improved drug characterizations.

Let $s_i$ denote a SMILES string. This SMILES string is input into two BERT models, yielding two different output vectors $h_i^z$ and $h_i^{z'}$ with different dropout masks. The two embeddings of the same SMILES string are treated as positive pairs, and other embeddings are selected as negative samples. The training objective for $h_i^z$ and $h_i^{z'}$ for the mini-batch number *N* of pairs is:

1 $$\begin{aligned} \ell _{i}=-\log \frac{e^{sim\left( {\textbf{h}}_{i}^{z_{i}}, {\textbf{h}}_{i}^{z_{i}^{\prime }}\right) / \tau }}{\sum _{j=1}^{N} e^{sim\left( {\textbf{h}}_{i}^{z_{i}}, {\textbf{h}}_{j}^{z_{j}^{\prime }}\right) / \tau }} \end{aligned}.$$

The fine-tuned BERT model evaluates encodings of SMILES strings more effectively than the original BERT model; given a drug pair, the embeddings of corresponding SMILES after the fine-tuned BERT encoding can be expressed as ($x_i$, $x_j$), where $x_i \in {\mathbb {R}}^D$ and $x_j \in {\mathbb {R}}^D$.

### Dimensional alignment

The model input includes hashed atom pair molecular fingerprints of drugs, SMILES string encodings and cell line gene expressions. The hashed atom pair molecular fingerprint is a molecular representation that transforms molecules into series of bit strings. However, the various inputs have different dimensions, with some inputs having high dimensions. To reduce the calculation costs and ensure that all inputs have the same dimensions, we project the hashed atom pair fingerprints of a drug pair $f_A^{i}, f_B^{i}$, the gene expression of the cell line *z*, and the SMILES string encodings $x_A^{i}$ and $x_B^{i}$ to the same dimension. Given g($\cdot$) as a projection equation, the output can be computed as:

2 $$\begin{aligned} g(x) = Wx + b \end{aligned}.$$

In the equation, *W* is the weight, and *b* is the bias. On the basis of the above equation, the inputs can be projected as follows: $f_A^{i'}$ and $f_B^{i'}$ for the fingerprints, $z^{'}$ for the cell lines, and $x_A^{i'}$ and $x_B^{i'}$ for SMILES encodings.

### Dual fusion

Most prior models concatenated only the drug features and cell line information as input into the multilayer fully connected network; however, this approach does not capture the potential information within the concatenated features. To generate more informative representations, in the feature fusion block, we propose a double-view feature fusion mechanism that reweights the input feature representations at the bit and vector levels simultaneously. Given $$f_A^{i'}$$ and $$f_B^{i'} $$ as the fingerprint representations of the drug pairs, $$x_A^{i'}$$ and $$h_B^{i'}$$ as the SMILES features of the drug pairs, and $$ z^{'} $$ as the gene expression of the cell line, the input to the fusion mechanism is:

3 $$\begin{aligned} l_i = concat(f_A^{i'},f_B^{i'},z^{'},x_A^{i'},x_B^{i'}) \end{aligned}.$$

#### Multi-head attention mechanism

**Fig. 2:**
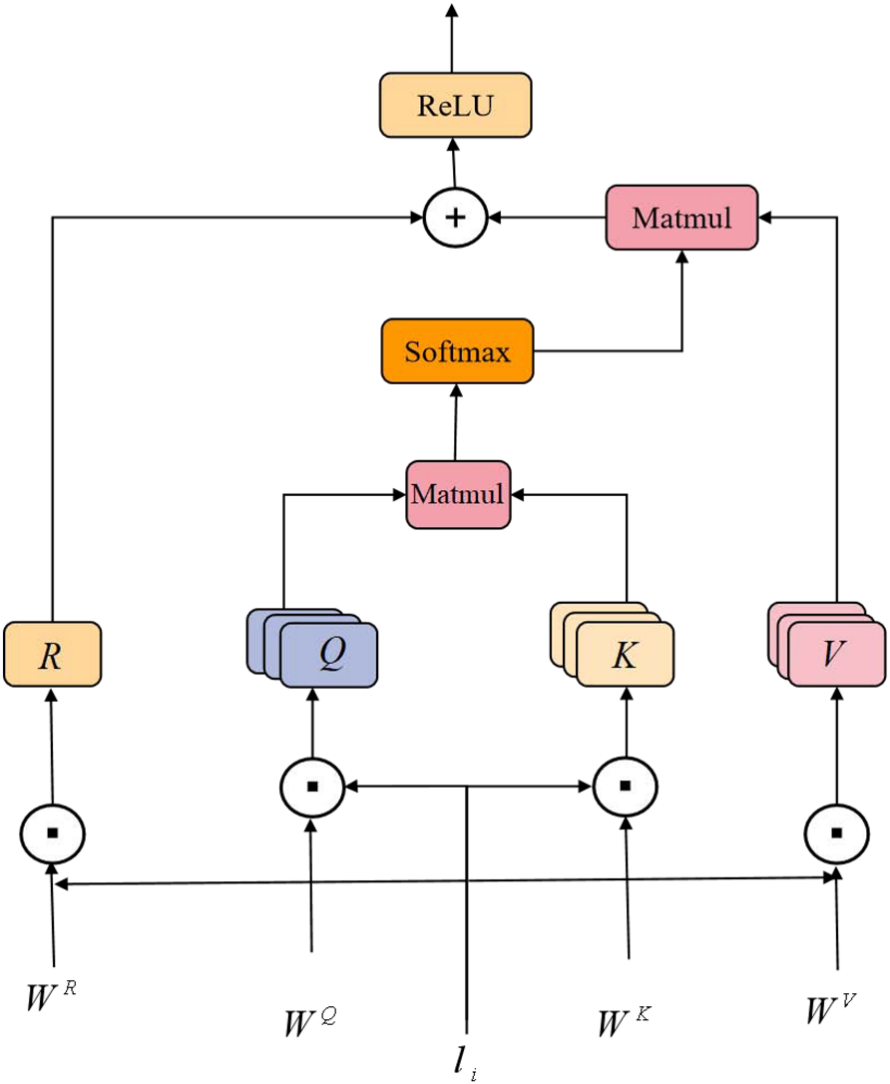
Multihead attention mechanism

Figure 2 shows the architecture of the multi-head attention mechanism. The attention module is utilized to capture interactions between features at the vector level. The important operation of the multi-head attention mechanism is the function *Attention*(*Q*, *K*, *V*), which takes three feature matrices ($Q \in R^{l_q \times d_k}$, $K \in R^{l_k \times d_k}$, and $V \in R^{l_v \times d_v}$) as inputs, where $l_q$, $l_k$ and $l_v$ are the dimensions of the input length and $d_k$ and $d_v$ indicate the transformed dimensions. Let $l_i$ be the input to the multi-head attention mechanism. Then, the output matrix can be obtained as follows:

4 $$\begin{aligned}{} & {} Q_i = l_i{W{i}^Q} \end{aligned},$$

5 $$\begin{aligned}{} & {} K_i = l_i{W{i}^K} \end{aligned},$$

6 $$\begin{aligned}{} & {} V_i = l_i{W{i}^V} \end{aligned},$$

7 $$\begin{aligned}{} & {} Attention(Q_i,K_i,V_i)=softmax(\frac{Q{K}^T}{\sqrt{d_k}})V \end{aligned},$$

where $W^K$, $W^V$, and $W^Q$ are weight matrices. $W^K$, $W^V$, and $W^Q$ are 2-dimensional matrices, and the 2 dimensions are the embedded size. The multi-head attention mechanism contains *h* heads, where the *i*-th head can be computed as:

8 $$\begin{aligned} M_i = Attention(Q_i,K_i,V_i) \end{aligned}.$$

Although previous experiments indicate that the expressiveness of a network increases with increasing network depth, it is wrong to interpret this result as the deeper the network is, the better the result [36]. As the number of network layers increases, the error increases. To alleviate the difficulty of training deep networks and reduce the training error, a residual block was added to the attention network. In the equation, $W^R$ is the parameter. $W^R$ is a 2-dimensional matrix, where the dimensions represent the embedded size. After the residual learning block, the ReLU activation function is performed, which can be computed as:

9 $$\begin{aligned} R = l_i{W^R} \end{aligned},$$

10 $$\begin{aligned} m_{vec}= M_i + R \end{aligned},$$

11 $$\begin{aligned} m_{vec} = ReLU(m_{vec}) \end{aligned}.$$

The output of the attention module is $m_{vec}$.

#### Highway network

In traditional deep learning, highway networks allow unimpeded information to flow across several layers. As the number of network layers increases, the network becomes more difficult to optimize. Highway networks have been used to partially address this optimization problem and prevent vanishing gradients. In the proposed model, the highway network learns feature information at the bitwise level. The input to the highway network module is $l_i$. The highway layer can be formulated as follows:

12 $$\begin{aligned} m_{bit} =g\odot t(l_i)+ (1-g)\odot q(l_i) \end{aligned}.$$

**Fig. 3:**
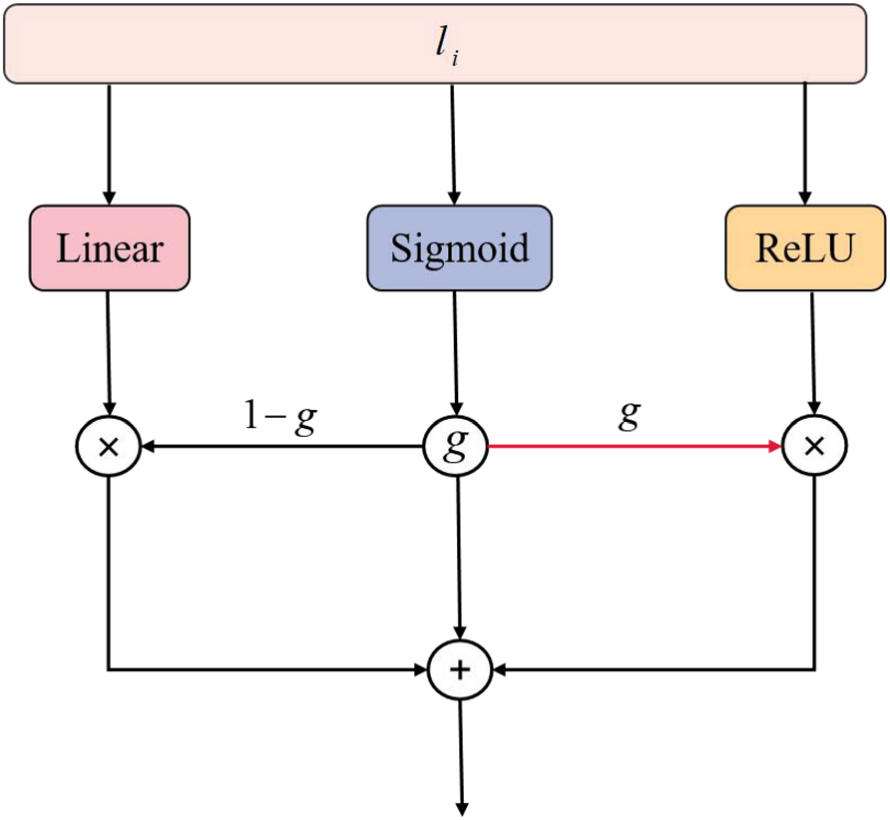
Highway network

In the above formula, $$t(l_i)$$ denotes a nonlinear transformation, which is the ReLU function in our experiments; $$g=\sigma (l_i)$$ is a sigmoid gate; $$q_i=linear(l_i) $$ is a linear transformation; $$(1-g)$$ is the carry gate; and $$m_{bit} $$ is the output of the highway network. Figure 3 shows the components of the highway network.

### Predicting the synergistic effect

The fusion features (which include the vector and bit levels) can be represented as:

13 $$\begin{aligned} m_x = m_{vec} + m_{bit} \end{aligned}.$$

The output of the model is the synergistic prediction score of the drug pair, which can be calculated as:

14 $$\begin{aligned} {{\hat{y}}} = LayerNorm(m_x) \end{aligned}.$$

Let ${\hat{y}}$ represent the synergistic prediction score of a drug pair, *y* is the real score. Then, the cross-entropy loss is adopted as the loss function to train the model, which is defined as:

15 $$\begin{aligned} L_{c}^i = -[y_{i}log \hat{y_i} + (1-y_{i})log(1- \hat{y_i})] \end{aligned}.$$

Given a sample, each input $l_i$ is passed through the network twice, resulting in two different output predictions, ${\hat{y}}_1^{i}$ and ${\hat{y}}_2^{i}$. Since the dropout mechanism randomly discards some neurons during each pass, ${\hat{y}}_1^{i}$ and ${\hat{y}}_2^{i}$ represent different prediction probabilities generated by two distinct subnets. Regularized dropout (R-drop) is applied to regularize the output predictions by minimizing the Kullback–Leibler (KL) divergence between two output distributions, which can be calculated as follows:

16 $$\begin{aligned}{} & {} D_{K L}({\hat{y}}_1^{i} \parallel {\hat{y}}_2^{i} )=\sum _{i=1}^N\left[ \left( {\hat{y}}_1^{i}\right) \log \left( {\hat{y}}_1^{i}\right) -\left( {\hat{y}}_1^{i}\right) \log \left( {\hat{y}}_2^{i}\right) \right], \end{aligned}$$

17 $$\begin{aligned}{} & {} L^i_{KL} = 1/2 (D_{KL}({\hat{y}}_1^{i}\parallel {\hat{y}}_2^{i}))+D_{KL}(({\hat{y}}_2^{i}\parallel {\hat{y}}_1^{i})). \end{aligned}$$

Moreover, the predictions ${\hat{y}}_1^{i}$ and ${\hat{y}}_2^{i}$ are both considered in the cross entropy loss by averaging their sum:

18 $$\begin{aligned} L_{cross}^i = 1/2 ( L_{c}^i({\hat{y}}_1^i)+ L_{c}^i({\hat{y}}_2^i)) \end{aligned}.$$

The final loss is calculated as:

19 $$\begin{aligned} L^i = L_{cross}^i + \alpha \cdot L^i_{KL} \end{aligned}.$$

In the above equation, $\alpha$ is the parameter.

## Results

To evaluate the experimental performance of our model, we compared our model with several competitive deep learning methods, including DeepDDS [24], EPGCNDS [26], GCNBMP [27], DeepSynergy [5], MatchMaker [25] and MRGNN [37]. To clarify the differences between our model and the above deep learning-based methods, we summarize the comparison methods below.

- DeepSynergy: DeepSynergy uses molecular chemistry and cell line genomic information as input and a deep neural network (DNN) to simulate drug synergy and predict the synergy score.
- MRGNN: MRGNN uses a multiresolution-based architecture to extract node features from neighborhoods of graph nodes, applies dual graph-state long short-term memory (LSTM) networks to summarize the local features of each graph, extracts interactions between pairwise graphs, and combines the results to predict the synergy score.
- GCNBMP: GCNBMP uses a Siamese GCN architecture to transform irregularly structured molecular data into real-valued embedding vectors, which are then input into an interaction predictor based on the HOLE-style neural network to predict interactions between the input drug pairs.
- EPGCN-DS: EPGCN-DS uses twin GCN branches to learn atom-level features. The drug is indicated as the sum of all atom features. The interaction decoder outputs the possibility of two drugs interacting with one another.
- DeepDDS: DeepDDS uses a graph neural network and attention mechanism to identify drug combinations, and its inputs are drug molecule structures and gene expression levels.
- MatchMaker: MatchMaker trains two parallel subnetworks to learn specific representations: the first subnetwork is for the drug structures, and the second is for the gene expression of the cell lines. The joint representation is then input into a third subnetwork to predict drug pair synergy.

The DeepSynergy, MRGNN and MatchMaker models predict continuous synergy scores. To compare with other methods, we converted the models into a classifier by transforming the last layer into a sigmoid function and changing the MSE Loss to CrossEntropyLoss. The hyperparameter settings of the compared methods were taken from Additional file 1: Table S9.

**Fig. 4:**
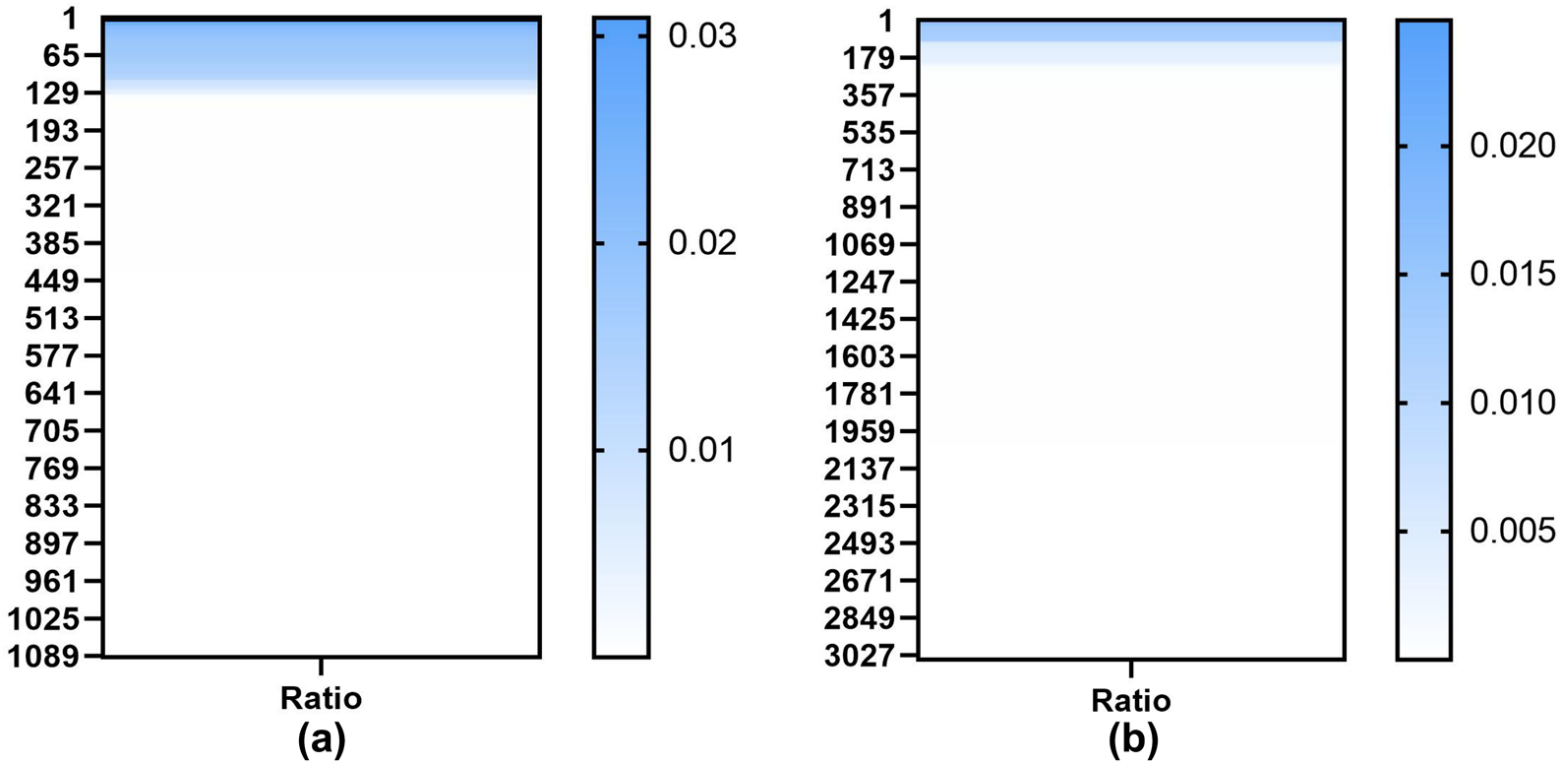
**a** Number of drug occurrences in the DrugcombDB database. **b** Number of drug occurrences in the Drugcomb database

### Dataset summary

We evaluated the performance of the models on two datasets, DrugComb [28] and DrugCombDB [29]. DrugComb is a network-based dataset that was released in 2019 and updated in March 2021 [38]. DrugComb provides experimental data on 739,964 drug combinations for 4268 drugs tested in 288 cell lines. DrugCombDB is a drug combination dataset that was released in 2019. DrugCombDB includes 498,865 drug combinations of 5350 drugs tested in 104 cell lines. The gene expression profiles were downloaded from the Cancer Cell Line Encyclopedia (CCLE) database [39], which contains the expression profiles of 1035 cell lines, covering 72 cell lines in DrugCombDB and 98 cell lines in DrugComb. After adding the gene expression profiles, the DrugCombDB dataset includes 106,709 combined experiments of 1084 drugs, and the DrugComb dataset includes 292,005 combined experiments of 3038 drugs. Figure 4 shows the number of drug occurrences in the DrugCombDB and DrugComb datasets. The figure shows that the 2 datasets are imbalanced; 11% of the drugs in DrugComb appear more than 300 times, and 7% of the drugs in DrugCombDB appear more than 200 times.

### Evaluation metrics

Nine metrics are used to measure the performance, including the accuracy (ACC), area under the receiver operator characteristics curve (ROC-AUC), balanced accuracy score (BACC), Matthews corrcoef (MCC), F1 score, recall (Rec), average precision (AP), precision (Prec) and kappa coefficient. These evaluation metrics are calculated as follows:

20 $$\begin{aligned}{} & {} ACC =\frac{TP+TN}{TP+TN+FP+FN}, \end{aligned}$$

21 $$\begin{aligned}{} & {} Precision=\frac{TP}{TP+FP}, \end{aligned}$$

22 $$\begin{aligned}{} & {} Recall=\frac{TP}{TP+FN}, \end{aligned}$$

23 $$\begin{aligned}{} & {} F1=\frac{2PR}{P+R}=\frac{2TP}{2TP+FP+FN}, \end{aligned}$$

24 $$\begin{aligned}{} & {} \textrm{MCC}=\frac{TP \times TN-FP\times FN}{\sqrt{(TP+FP)(TP+FN)(TN+FP)(TN+FN)}}, \end{aligned}$$

25 $$\begin{aligned}{} & {} \textrm{AP}=\sum _{n}\left( R_{n}-R_{n-1}\right) P_{n}, \end{aligned}$$

26 $$\begin{aligned}{} & {} Kappa=\frac{P_{o}-P_{e}}{1-P_{e}}, \end{aligned}$$

27 $$\begin{aligned}{} & {} BACC= \frac{TPR+TNR}{2}, \end{aligned}$$

28 $$\begin{aligned}{} & {} TPR = \frac{TN}{TN+FP}. \end{aligned}$$

In the equations, TP denotes true positives, TN denotes true negatives, FP denotes false positives, and FN denotes false negatives. The balanced accuracy score is used to handle imbalanced datasets and is defined as the average recall score obtained in each class. TPR represents the recall score, and TNR is the recognition rate (coverage rate) of the model for negative samples. The MCC is mainly used to evaluate binary classification problems and is a relatively balanced metric. Kappa is a consistency measure; in this case, consistency indicates whether the model prediction results are consistent with the actual classification results. $P_o$ denotes the accuracy, assuming that the number of real samples in each class is $a_{1}, a_{2},..., a_{c}$, the number of predicted samples in each class is $b_{1}, b_{2},..., b_{c}$, and the total number of samples is *n*. $P_e$ is calculated as

29 $$\begin{aligned} P_e = \frac{a_{1} \times b_{1} +a_{2}\times b_{2}+...+a_{c}\times b_{c}}{n\times n} \end{aligned}.$$

### Experimental settings

First, we conducted a 5-fold cross-validation to evaluate the predictive power of DFFNDDS. The training samples are randomly divided into five subsets of approximately equal size; every four subsets are treated as training datasets, while the one left is used as the test set. The average prediction accuracy over the 5-fold cross-validation is used as the final performance measure. Under the random splitting setting, the ratio of synergistic/antagonistic pairs in 5 cross-validations is the same. In the DrugcombDB dataset, the ratio of synergistic/antagonistic pairs is 0.4, in the Drugcomb dataset, the ratio is 1.5.

To verify the prediction performance of DFFNDDS, we used leave-one-out cross-validation. First, leave-one-drug-combination-out cross-validation is used to evaluate the performance of predicting unlearned drug combinations. This method iteratively excludes drug pairs from the training set and uses the remaining drug combinations as the test set.

However, drug combinations alone cannot exclude single drugs from the training set, and the same drug may be used in both the training and testing sets. Thus, the next division method is to leave one drug out to verify the ability of the model to learn features of unseen drugs based on the chemical structures of known drugs.

In addition, leave-one-cell-line-out experiments are implemented to verify the performance of DFFNDDS. We excluded all cell lines in the training set and used the excluded data as the test set to ensure that the model did not know the gene expression of the excluded cell lines. This method is applied to assess the ability of the model to predict drug synergy scores in unknown environments. The ratios of synergistic/antagonistic in cross-validation under different leave-one-out experiments in two datasets are discussed in Additional file 1: Tables S1–S8. In the different splitting settings, despite the influence of uneven drug distribution, the ratio of synergistic/antagonistic is similar in different splitting settings.

### Performance evaluation

We binarized the predictive probability with a threshold of 0.5. Tables 1 and 2 summarize the performance measures of DFFNDDS and the comparison methods on the different datasets. Table 1 shows that our method demonstrated the best overall performance. In terms of the ACC score, DFFNDDS achieved a value of 0.871, demonstrating higher accuracy than all other methods. In terms of the Prec, Rec, and F1 scores, DFFNDDS achieved the best scores on the DrugCombDB dataset, with values of 0.801, 0.746, and 0.773, respectively. The results show that DFFNDDS clearly recognized synergistic drug combinations. To prevent the imbalanced datasets from impacting the model evaluation results, we used the BACC, MCC and Kappa metrics. The table shows that our proposed method achieved BACC, MCC and Kappa scores of 0.834, 0.684, and 0.683, respectively. To comprehensively evaluate the method, the AUC and AP metrics were used. DFFNDDS achieved ROC-AUC and AP values of 0.921 and 0.859, respectively. Thus, the 9 performance metrics show various aspects of the model performance.

Table 2 shows that the models perform worse on the DrugComb dataset than on the DrugCombDB dataset; however, our method still achieved better performance than the other approaches on 8 of the 9 metrics. Table 2 shows that DFFNDDS achieved ACC, Prec, Rec, and F1 scores of 0.768, 0.788, 0.840, and 0.813, respectively. DFFNDDS exhibited slightly worse performance than GCNBMP, DeepDDS, EPGCNDS and MatchMaker in terms of the Recall score. However, we consider the F1 score, which is a weighted average of the precision and recall that reflects the robustness of a model. DFFNDDS achieved a better F1 score than the comparison methods. In terms of the ability of DFFNDDS to handle imbalanced datasets, our proposed model showed competitive performance, with BACC, MCC and Kappa scores of 0.749, 0.509, and 0.507, respectively. Moreover, DFFNDDS demonstrated the best performance in terms of the ROC-AUC and AP metrics, with values of 0.846 and 0.890, respectively. Furthermore, in general, DFFNDDS has lower standard deviations than the other methods on the considered performance metrics. Therefore, the fivefold cross-validation results show the competitiveness of our proposed method.

Regarding the leave-one-out cross-validation results, in the leave-one-drugpairs-out experiments on two datasets, Tables 7 and 8 showed the results on two datasets. DFFNDDS achieved the best scores on all performance metrics on the Drugcomb dataset, the model performed the best in 4 metrics and maintained the top 3 performance compared to baselines in the other 5 metrics. For the leave-one-cell-line-out experiments on the DrugComb dataset, Tables 4 and 6 display the performance. In the DrugcomDB, DFFNDDS achieved the best scores of 7 in 9 metrics, especially in ROC-AUC and MCC metrics, DFFNDDS outperformed other methods by 17%. In the Drugcomb dataset, DFFNDDS performed the second best in the metrics, which is only a little inferior to MRGNN. For the leave-one-drug-out experiments, our model did not achieve state-of-the-art performance in terms of the Recall metric on the DrugCombDB or DrugComb datasets; however, our model has superior results on at least 5 metrics, as shown in Tables 3 and 5. The reason for these results might be that our model classifies more synergistic drug combinations as antagonist drug combinations. These results which used every single observation in the dataset prove the robustness of the DFFNDDS model, it maintained the top3 performance under all the leave-one-out splitting settings. From these results of Tables, we are concerned that our proposed method DFFNDDS has competitive performance compared to baselines.

**Table 1:** Performance comparison of DFFNDDS and competitive methods on the DrugCombDB dataset under random splits

Method	ACC	BACC	Prec	Rec	F1	ROC AUC	MCC	Kappa	AP
DFFNDDS	**0.871(0.002)**	**0.834(0.002)**	**0.801(0.008)**	**0.746(0.006)**	**0.773(0.003)**	**0.921(0.003)**	**0.684(0.004)**	**0.683(0.004)**	**0.859(0.005)**
DeepDDS	0.852(0.002)	0.784(0.004)	0.783 (0.010)	0.675 (0.011)	0.725 (0.005)	0.900 (0.004)	0.624 (0.006)	0.621 (0.006)	0.821 (0.007)
DeepSynergy	0.827(0.003)	0.762(0.005)	0.760(0.010)	0.604 (0.011)	0.673 (0.009)	0.860(0.002)	0.565 (0.010)	0.558(0.010)	0.773(0.008)
MRGNN	0.810(0.003)	0.737(0.006)	0.730(0.010)	0.560 (0.017)	0.634(0.008)	0.845(0.001)	0.517 (0.007)	0.508(0.008)	0.734(0.004)
GCNBMP	0.802(0.002)	0.717(0.006)	0.737(0.014)	0.511(0.018)	0.603(0.010)	0.828(0.005)	0.492(0.006)	0.477(0.007)	0.710(0.007)
EPGCNDS	0.754(0.003)	0.630(0.013)	0.672(0.028)	0.328 (0.039)	0.438(0.031)	0.740(0.002)	0.338(0.012)	0.305(0.022)	0.583(0.007)
MatchMaker	0.762(0.005)	0.638(0.009)	0.698(0.006)	0.339(0.003)	0.454(0.002)	0.746(0.007)	0.360(0.012)	0.325(0.005)	0.568(0.002)

The best performing model for each dataset and metric is highlighted in bold

**Table 2:** Performance comparison of DFFNDDS and competitive methods on the DrugComb dataset under random splits

Method	ACC	BACC	Prec	Rec	F1	ROC AUC	MCC	Kappa	AP
DFFNDDS	**0.768 (0.002)**	**0.749(0.004)**	**0.788(0.006)**	0.840(0.009)	**0.813(0.002)**	**0.846(0.002)**	**0.509(0.005)**	**0.507(0.006)**	**0.890(0.001)**
DeepDDS	0.745 (0.003)	0.710(0.005)	0.743(0.006)	0.881 (0.007)	0.806(0.001)	0.825(0.002)	0.455(0.005)	0.441 (0.008)	0.876 (0.001)
DeepSynergy	0.719(0.001)	0.692(0.003)	0.739 (0.006)	0.826(0.009)	0.780(0.002)	0.790(0.003)	0.401(0.003)	0.396(0.005)	0.849 (0.003)
MRGNN	0.665(0.003)	0.631(0.002)	0.693(0.001)	0.798(0.011)	0.742(0.005)	0.716(0.003)	0.279(0.005)	0.273(0.004)	0.795(0.003)
GCNBMP	0.628(0.033)	0.551(0.012)	0.635(0.013)	**0.930(0.008)**	0.750(0.001)	0.587(0.006)	0.211(0.006)	0.237(0.001)	0.679(0.006)
EPGCNDS	0.628(0.004)	0.572(0.003)	0.645(0.004)	0.850(0.008)	0.734(0.004)	0.647(0.004)	0.173(0.008)	0.156(0.007)	0.733(0.005)
MatchMaker	0.695(0.004)	0.649(0.007)	0.697(0.005)	0.874(0.008)	0.776(0.002)	0.757(0.004)	0.340 (0.010)	0.319(0.010)	0.824(0.003)

The best performing model for each dataset and metric is highlighted in bold

**Table 3:** Performance comparison of DFFNDDS and competitive methods on the DrugCombDB dataset under leave-one-drug-out splits

Method	ACC	BACC	Prec	Rec	F1	ROC AUC	MCC	Kappa	AP
DFFNDDS	0.681(0.046)	**0.599(0.028)**	**0.512(0.128)**	0.360(0.052)	**0.416(0.062)**	**0.651(0.042)**	**0.216(0.058)**	**0.205(0.052)**	**0.489(0.125)**
DeepDDS	0.689(0.024)	0.586(0.024)	0.433(0.066)	0.349(0.060)	0.380(0.033)	0.621(0.030)	0.184 (0.046)	0.179(0.044)	0.400(0.040)
DeepSynergy	0.697(0.026)	0.588(0.022)	0.470(0.091)	0.324(0.090)	0.369(0.053)	0.630(0.034)	0.199(0.032)	0.187 (0.033)	0.450 (0.071)
MRGNN	0.622(0.028)	0.557(0.021)	0.361(0.033)	**0.404(0.035)**	0.379(0.020)	0.574(0.031)	0.111(0.041)	0.110(0.041)	0.342 (0.027)
GCNBMP	0.684(0.019)	0.556(0.035)	0.332(0.168)	0.254(0.138)	0.286 (0.148)	0.585 (0.051)	0.122(0.073)	0.120(0.072)	0.373(0.044)
EPGCNDS	0.678(0.008)	0.580(0.003)	0.418(0.007)	0.364(0.007)	0.379(0.003)	0.631(0.003)	0.170(0.004)	0.165 (0.007)	0.395 (0.008)
MatchMaker	** 0.732(0.025)**	0.586(0.023)	0.479(0.066)	0.280(0.064)	0.351(0.066)	0.649(0.018)	0.209(0.047)	0.197(0.047)	0.415(0.072)

The best performing model for each dataset and metric is highlighted in bold

**Table 4:** Performance comparison of DFFNDDS and competitive methods on the DrugCombDB dataset under leave-one-cellline-out splits

Method	ACC	BACC	Prec	Rec	F1	ROC AUC	MCC	Kappa	AP
DFFNDDS	**0.799(0.008)**	**0.731(0.010)**	0.677(0.028)	0.572(0.028)	**0.619(0.013)**	**0.829(0.010)**	**0.489(0.017)**	**0.485(0.017)**	**0.693(0.018)**
DeepDDS	0.790(0.009)	0.713(0.009)	0.675(0.037)	0.531(0.017)	0.593(0.016)	0.812 (0.007)	0.461(0.023)	0.455(0.021)	0.676 (0.024)
DeepSynergy	0.583(0.004)	0.629 (0.015)	0.393(0.003)	**0.743(0.017)**	0.513(0.018)	0.630(0.034)	0.199(0.032)	0.187 (0.033)	0.450 (0.071)
MRGNN	0.788(0.010)	0.692(0.016)	**0.723(0.036)**	0.458(0.049)	0.558(0.03)	0.806(0.007)	0.449(0.012)	0.428 (0.023)	0.676(0.02)
GCNBMP	0.673(0.190)	0.620(0.098)	0.491(0.095)	0.488(0.017)	0.437(0.024)	0.688(0.054)	0.275(0.025)	0.265(0.017)	0.531(0.091)
EPGCNDS	0.745 (0.007)	0.613(0.005)	0.667 (0.025)	0.288(0.019)	0.401(0.015)	0.727(0.015)	0.309(0.009)	0.270(0.008)	0.566(0.014)
MatchMaker	0.680(0.027)	0.650(0.012)	0.463 (0.053)	0.575(0.037)	0.509(0.021)	0.701(0.018)	0.284(0.032))	0.279(0.037)	0.505(0.048)

The best performing model for each dataset and metric is highlighted in bold

**Table 5:** Performance comparison of DFFNDDS and competitive methods on the DrugComb dataset under leave-one-drug-out splits

Method	ACC	BACC	Prec	Rec	F1	ROC AUC	MCC	Kappa	AP
DFFNDDS	**0.592(0.028)**	0.529(0.012)	**0.628(0.041)**	0.823(0.049)	0.711(0.028)	**0.560(0.016)**	**0.081(0.028)**	0.062(0.026)	**0.671(0.035)**
DeepDDS	0.587(0.009)	0.527(0.004)	0.642(0.007)	0.768(0.038)	0.699(0.014)	0.550(0.010)	0.061(0.010)	0.057(0.008)	0.666(0.009)
DeepSynergy	0.566(0.014)	0.528(0.013)	0.610(0.017)	0.732(0.041)	0.665 (0.023)	0.550(0.019)	0.060(0.018)	0.058(0.019)	0.632(0.025)
MRGNN	0.563(0.018)	0.505(0.003)	0.609(0.021)	0.786(0.072)	0.684(0.028))	0.518(0.008)	0.011(0.007)	0.010(0.007)	0.623(0.022)
GCNBMP	0.474 (0.09)	0.501(0.002)	0.243(0.298)	0.353(0.439)	0.287(0.352)	0.507(0.015)	0.003 (0.005)	0.002(0.005)	0.610(0.021)
EPGCNDS	0.590(0.024)	0.511(0.008)	0.617(0.019)	**0.868(0.086)**	**0.720(0.032)**	0.529 (0.017)	0.037(0.031)	0.024(0.018)	0.634(0.036)
MatchMaker	0.583(0.005)	**0.530(0.011)**	0.604(0.010)	0.838(0.042)	0.701(0.012)	0.569(0.016)	0.076(0.019)	**0.066(0.021)**	0.648(0.014)

The best performing model for each dataset and metric is highlighted in bold

**Table 6:** Performance comparison of DFFNDDS and competitive methods on the DrugComb dataset under leave-one-cellline-out splits

Method	ACC	BACC	Prec	Rec	F1	ROC AUC	MCC	Kappa	AP
DFFNDDS	0.635(0.014)	0.605(0.015)	0.662(0.02)	0.783(0.023)	0.717(0.011)	0.671(0.019)	0.224(0.026)	0.217(0.030)	0.745(0.027)
DeepDDS	0.638(0.004)	0.590(0.002)	0.659(0.006)	0.825(0.009)	0.733(0.006)	0.651(0.008)	0.204(0.006)	0.192(0.005)	0.722 (0.018)
DeepSynergy	0.606(0.006)	0.564(0.017)	0.628(0.009)	0.807(0.034)	0.706(0.010)	0.609(0.011)	0.147 (0.032)	0.136(0.033)	0.679(0.012)
MRGNN	**0.648(0.010)**	**0.619(0.009)**	**0.685(0.014)**	**0.765(0.010)**	**0.723(0.011)**	**0.694(0.014)**	**0.248(0.019)**	**0.245(0.019)**	**0.775(0.018)**
GCNBMP	0.623(0.024)	0.565(0.023)	0.651(0.012)	0.732(0.022)	0.730(0.022)	0.607 (0.017)	0.134(0.010)	0.132(0.008)	0.702(0.025)
EPGCNDS	0.620(0.010)	0.559(0.005)	0.649(0.013)	0.829(0.03)	0.728(0.014)	0.625(0.008)	0.139(0.006)	0.128(0.008)	0.725(0.016)
MatchMaker	0.609(0.006)	0.537(0.014)	0.632(0.015)	0.867(0.071)	0.729(0.019)	0.574(0.018)	0.100(0.022)	**0.082(0.028)**	0.664(0.019)

The best performing model for each dataset and metric is highlighted in bold

**Table 7:** Performance comparison of DFFNDDS and competitive methods on the Drugcomb dataset under leave-one-drugpairs-out splits

Method	ACC	BACC	Prec	Rec	F1	ROC AUC	MCC	Kappa	AP
DFFNDDS	**0.737(0.0008)**	**0.713(0.003)**	**0.755(0.005)**	**0.831(0.010)**	**0.792(0.004)**	**0.810(0.003)**	**0.441(0.004)**	**0.437(0.005)**	**0.859(0.002)**
DeepDDS	0.720(0.003)	0.684(0.006)	0.728(0.004)	0.857(0.014)	0.787(0.004)	0.792(0.006)	0.398(0.009)	0.387(0.010)	0.852 (0.002)
DeepSynergy	0.703 (0.003)	0.672(0.004)	0.727(0.008)	0.817(0.021)	0.769(0.006)	0.768(0.002)	0.362(0.004)	0.356(0.005)	0.833(0.003)
MRGNN	0.635 (0.003)	0.600(0.004)	0.670(0.005)	0.772(0.017)	0.717(0.005)	0.661(0.004)	0.213(0.006)	0.209(0.006)	0.740(0.004)
GCNBMP	0.619(0.008)	0.571(0.036)	0.651(0.023)	0.807(0.019)	0.716(0.021)	0.621(0.061)	0.151(0.076)	0.148(0.074)	0.711 (0.053)
EPGCNDS	0.626(0.006)	0.570(0.004)	0.643(0.005)	0.845(0.028)	0.731(0.011)	0.641(0.005)	0.168 (0.006)	0.151(0.007)	0.726(0.006)
MatchMaker	0.684(0.003)	0.638(0.005)	0.689(0.008)	0.866(0.016)	0.767(0.002)	0.738(0.002)	0.314(0.005)	0.294(0.009)	0.807(0.003)

The best performing model for each dataset and metric is highlighted in bold

**Table 8:** Performance comparison of DFFNDDS and competitive methods on the DrugcombDB dataset under leave-one-drugpairs-out splits

Method	ACC	BACC	Prec	Rec	F1	ROC AUC	MCC	Kappa	AP
DFFNDDS	0.739(0.002)	0.718(0.002)	**0.759(0.003)**	**0.825(0.009)**	**0.791(0.003)**	0.816(0.001)	0.447(0.003)	0.445(0.003)	**0.864(0.002)**
DeepDDS	**0.822(0.004)**	**0.749(0.008)**	0.756 (0.020)	0.575(0.017)	0.653(0.014)	**0.856(0.007)**	**0.545(0.015)**	**0.536(0.014)**	0.756(0.014)
DeepSynergy	0.795(0.006)	0.705(0.010)	0.730(0.008)	0.486(0.001)	0.583(0.006)	0.809(0.007)	0.471(0.016)	0.455(0.008)	0.693(0.001)
MRGNN	0.754(0.007)	0.670(0.009)	0.624 (0.017)	0.459(0.028)	0.528(0.019)	0.755(0.009)	0.376(0.016)	0.367(0.017)	0.606(0.017)
GCNBMP	0.652 (0.177)	0.576(0.093)	0.305(0.274)	0.315(0.260)	0.606(0.130)	0.161(0.198)	0.151(0.076)	0.160(0.196)	0.417(0.154)
EPGCNDS	0.750(0.003)	0.611(0.005)	0.704(0.004)	0.270(0.004)	0.389(0.007)	0.732(0.006)	0.319(0.004)	0.269(0.005)	0.573(0.013)
MatchMaker	0.805(0.003)	0.717(0.008)	0.738 (0.014)	0.508(0.018)	0.602(0.016)	0.822(0.007)	0.493(0.014)	0.479 (0.015)	0.708(0.017)

The best performing model for each dataset and metric is highlighted in bold

### Ablation analysis

We performed ablation analyses to investigate whether the inclusion of the attention mechanism, highway network, fine-tuned BERT model, and inputs improve the predictive performance of the model. To demonstrate the importance of each model component, we conducted ablation analyses by removing some model components. Specifically, we compared the DFFNDDS results of: (i) DFFNDDS without the attention mechanism, (ii) DFFNDDS without the highway network, (iii) DFFNDDS without SMILES string inputs, (iv) DFFNDDS without fingerprint inputs, and (v) DFFNDDS without the fine-tuned BERT. The comparison was performed based on 5-fold cross-validation tests on the training dataset. These results on the DrugCombDB dataset are summarized in Table 9.

The results revealed that the complete DFFNDDS framework achieves the best predictive performance on 8 of the 9 evaluation metrics. In contrast, DFFNDDS without fingerprints displayed the worst performance. The results demonstrated that fingerprint inputs and the highway network play important roles in ensuring high-quality drug synergy predictions. This may be because fingerprints contain considerable chemical information about drugs. The highway network contributes more to learning drug features than the attention mechanism. The attention mechanism might not capture as much SMILES information as expected. In terms of model design, the ablation experiments indicated that combining fingerprint inputs and SMILES strings is effective. The DFFNDDS models without the attention mechanism and highway network performed worse than DFFNDDS, which indicates that the attention mechanism and highway network enhance the performance of DFFNDDS, possibly due to the complementarity of the features extracted by different feature extractors. Moreover, the results of the DeepChem encoding framework confirmed that the fine-tuned BERT model is indispensable.

Meanwhile, We also provided the results of DFFNDDS without R-drop loss. To explore the effect of the R-drop loss, we applied the R-drop on compared models, these results are discussed in Additional file 1: Table S10. Additional file 1: Table S10 shows that R-drop doesn’t enhance all the performance of the models, so we concluded that the real novelty that gives the performance improvement is the framework of the model.

**Table 9:** Ablation analysis on the DrugCombDB dataset

Method	ACC	BACC	Prec	Rec	F1	ROC AUC	MCC	Kappa	AP
DFFNDDS	**0.871(0.002)**	**0.835(0.002)**	** 0.801(0.008)**	0.747(0.006)	**0.773(0.004)**	** 0.922(0.003)**	**0.684(0.005)**	**0.683(0.005)**	**0.859(0.006)**
w.o. R-drop	0.866(0.002)	0.832(0.006)	0.785(0.011)	**0.750(0.017)**	0.767(0.007)	0.919(0.004)	0.674(0.007)	0.673(0.008)	0.854(0.007)
w.o. fingerprint	0.841 (0.035)	0.789 (0.056)	0.769 (0.050)	0.663 (0.107)	0.710 (0.086)	0.886 (0.054)	0.606 (0.099)	0.602 (0.103)	0.805 (0.086)
w.o.fine-tuned BERT	0.859 (0.009)	0.818 (0.005)	0.797 (0.016)	0.713 (0.017)	0.752 (0.004)	0.914 (0.002)	0.657 (0.003)	0.655 (0.003)	0.843 (0.004)
w.o. attention mechanism	0.860 (0.006)	0.819 (0.012)	0.794 (0.014)	0.719 (0.025)	0.754 (0.016)	0.913 (0.008)	0.659 (0.019)	0.657 (0.019)	0.838 (0.014)
w.o. highway network	0.857 (0.003)	0.816 (0.007)	0.785 (0.008)	0.714 (0.017)	0.747 (0.009)	0.914 (0.003)	0.649 (0.009)	0.648 (0.011)	0.842 (0.008)
w.o. SMILES	0.858 (0.005)	0.815 (0.009)	0.791 (0.007)	0.709 (0.019)	0.748 (0.012)	0.912 (0.002)	0.651 (0.013)	0.649 (0.014)	0.841 (0.008)

The best performing model for each dataset and metric is highlighted in bold

## Discussion

From the results, though our model performed significantly better than other methods, the performance in 9 metrics reflected that our model is still limited. The performance might be due to the features of drugs and information of cell lines haven’t been researched and dug thoroughly in the model. Another contributing factor may be the network, we suspect the network we chose doesn’t fit the prediction of drug combinations entirely. We believe that the model can be enhanced by feeding into more effective representations of drugs and information about cell lines, the more appropriate networks are considered in the enhancement, too. On the other hand, the results in leave-one-out cross-validation concern that our model has poor performance in generalization ability. But in reality, the leave-one-out cross-validation is more commonly used as we need to identify unfamiliar drug combinations inevitably. To solve the problem, we recommend trying transfer learning and other advanced machine learning to enhance the performance in leave-one-out cross-validation.

## Conclusions

In this paper, we proposed DFFNDDS, a novel model for predicting the synergy scores of drug combinations. In the model, the cell line information is represented by gene expression, and the drugs are represented by SMILES strings and fingerprints. we presented SMILES strings pretraining with fine-tuned BERT model and fused all the features not only at the bit-wise level but also at the vector-wise level. Compared to other competitive methods, DFFNDDS achieved state-of-the-art performance on the DrugComb and DrugCombDB datasets. Moreover, DFFNDDS outperformed other methods in terms of most evaluation metrics in strict leave-one-out cross-validation experiments. Overall, our method provides a new tool for identifying synergistic drug combinations.

## Supplementary Information

**Additional file 1.** Additional tables for DFFNDDS.

## Data Availability

Our datasets and code are publicly available at GITHUB via https://github.com/sorachel/DFFNDDS.
