# Supplementary material for: DFFNDDS: prediction of synergistic drug combinations with dual feature fusion networks
Source: J Cheminform. 2023 Mar 16;15:33. doi: 10.1186/s13321-023-00690-3 (PMC10022091; doi:10.1186/s13321-023-00690-3)
Supplement: Supplementary file 1 — Additional file 1. Additional tables for DFFNDDS. [file 13321_2023_690_MOESM1_ESM.docx]

Additional Tables for “DFFNDDS: PREDICTION OF SYNERGISTIC DRUG COMBINATIONS WITH DUAL FEATURE FUSION NETWORKS”

Mengdie Xu, Xinwei Zhao, Jingyu Wang, Wei Feng, Naifeng Wen, Chunyu Wang, Junjie Wang, Yun Liu* and Lingling Zhao*

**Additional tables**

Table S1–S8 contains the ratio of Synergistic/Antagonistic combinations on Drugcomb and DrugcombDB datasets under different splitting settings. Table S9 shows the hyperparamater settings in the compared methods. Table S10 is the results of compared methods with r-drop and without R-drop in DrugcombDB.

Table S1 Ratio of Synergistic/Antagonistic combinations on DrugcombDB under Random splitting setting

|  | Cross-validation1 | | Cross-validation2 | | Cross-validation3 | | Cross-validation4 | | Cross-validation5 | |
| --- | --- | --- | --- | --- | --- | --- | --- | --- | --- | --- |
| Training set | 24921/60446 | 0.4 | 24827/60540 | 0.4 | 24953/60414 | 0.4 | 24841/60526 | 0.4 | 24960/60407 | 0.4 |
| Validation set | 3116/7555 | 0.4 | 3212/7459 | 0.4 | 3107/7564 | 0.4 | 3163/7508 | 0.4 | 3086/7585 | 0.4 |
| Testing set | 3128/7543 | 0.4 | 3126/7545 | 0.4 | 3105/7566 | 0.4 | 3161/7510 | 0.4 | 3119/7552 | 0.4 |

Table S2 Ratio of Synergistic/Antagonistic combinations on Drugcomb under Random splitting setting

|  | Cross-validation1 | | Cross-validation2 | | Cross-validation3 | | Cross-validation4 | | Cross-validation5 | |
| --- | --- | --- | --- | --- | --- | --- | --- | --- | --- | --- |
| Training set | 140622/92982 | 1.5 | 140639/92965 | 1.5 | 140737/92867 | 1.5 | 140634/92970 | 1.5 | 14606/92998 | 1.5 |
| Validation set | 17664/11537 | 1.5 | 17635/11566 | 1.5 | 17639/11562 | 1.5 | 17526/11675 | 1.5 | 17632/11569 | 1.5 |
| Testing set | 17530/11670 | 1.5 | 17542/11658 | 1.5 | 17440/11760 | 1.5 | 17656/11544 | 1.5 | 17578/11622 | 1.5 |

Table S3 Ratio of Synergistic/Antagonistic combinations on Drugcomb under leave one-drug-out splitting setting

|  | Cross-validation1 | | Cross-validation 2 | | Cross-validation 3 | | Cross-validation 4 | | Cross-validation 5 | |
| --- | --- | --- | --- | --- | --- | --- | --- | --- | --- | --- |
| Training set | 80253/52382 | 1.5 | 86769/58130 | 1.5 | 81094/54312 | 1.5 | 82684/56095 | 1.5 | 78187/52054 | 1.5 |
| Validation set | 26732/17480 | 1.5 | 28929/19371 | 1.5 | 27168/17968 | 1.5 | 27503/18757 | 1.5 | 26166/17248 | 1.5 |
| Testing set | 8269/5554 | 1.5 | 6286/3393 | 1.9 | 7563/5041 | 1.5 | 7285/4582 | 1.6 | 8947/5966 | 1.5 |

Table S4 Ratio of Synergistic/Antagonistic combinations on Drugcomb under leave-cell line-out splitting setting

|  | Cross-validation1 | | Cross-validation 2 | | Cross-validation 3 | | Cross-validation 4 | | Cross-validation 5 | |
| --- | --- | --- | --- | --- | --- | --- | --- | --- | --- | --- |
| Training set | 100956/75931 | 1.5 | 110799/72699 | 1.5 | 107912/68726 | 1.5 | 108658/70937 | 1.5 | 111993/75129 | 1.5 |
| Validation set | 36837/25459 | 1.5 | 37066/24100 | 1.5 | 35959/22921 | 1.5 | 36147/23718 | 1.5 | 37319/25055 | 1.5 |
| Testing set | 28023/14799 | 1.5 | 27951/19390 | 1.5 | 31945/24542 | 1.5 | 31011/21534 | 1.5 | 26504/16005 | 1.5 |

Table S5 Ratio of Synergistic/Antagonistic combinations on Drugcomb under leave-drugpairs-out splitting setting

|  | Cross-validation1 | | Cross-validation 2 | | Cross-validation 3 | | Cross-validation 4 | | Cross-validation 5 | |
| --- | --- | --- | --- | --- | --- | --- | --- | --- | --- | --- |
| Training set | 104992/69371 | 1.5 | 105267/69985 | 1.5 | 105069/69158 | 1.5 | 105178/69705 | 1.5 | 106108/70119 | 1.5 |
| Validation set | 34952/23169 | 1.5 | 35077/23341 | 1.5 | 34981/23095 | 1.5 | 34932/23363 | 1.5 | 35348/23395 | 1.5 |
| Testing set | 35872/23649 | 1.5 | 35472/22863 | 1.5 | 35766/23936 | 1.5 | 35706/23121 | 1.5 | 34360/22675 | 1.5 |

Table S6 Ratio of Synergistic/Antagonistic combinations on DrugcombDB under leave-drugpairs-out splitting setting

|  | Cross-validation1 | | Cross-validation 2 | | Cross-validation 3 | | Cross-validation 4 | | Cross-validation 5 | |
| --- | --- | --- | --- | --- | --- | --- | --- | --- | --- | --- |
| Training set | 18563/45604 | 0.4 | 18752/45391 | 0.4 | 18672/45555 | 0.4 | 18734/45223 | 0.4 | 18640/45673 | 0.4 |
| Validation set | 6128/15262 | 0.4 | 6232/15149 | 0.4 | 6289/15120 | 0.4 | 6217/15102 | 0.4 | 6274/15164 | 0.4 |
| Testing set | 6474/14678 | 0.4 | 6181/15004 | 0.4 | 6204/14869 | 0.4 | 6214/15219 | 0.4 | 6251/14707 | 0.4 |

Table S7 Ratio of Synergistic/Antagonistic combinations on DrugcombDB under leave-one-drug-out splitting setting

|  | Cross-validation1 | | Cross-validation 2 | | Cross-validation 3 | | Cross-validation 4 | | Cross-validation5 | |
| --- | --- | --- | --- | --- | --- | --- | --- | --- | --- | --- |
| Training set | 12988/29588 | 0.4 | 16687/44780 | 0.4 | 16140/42787 | 0.4 | 15346/37404 | 0.4 | 16076/40330 | 0.4 |
| Validation set | 4311/9881 | 0.4 | 5525/14965 | 0.4 | 5405/14238 | 0.4 | 5049/12535 | 0.4 | 5288/13515 | 0.4 |
| Testing set | 1910/6148 | 0.3 | 770/940 | 0.8 | 771/1146 | 0.7 | 1085/2539 | 0.4 | 980/1303 | 0.8 |

Table S8 Ratio of Synergistic/Antagonistic combinations on DrugcombDB under leave-cell-line-out setting

|  | Cross-validation1 | | Cross-validation 2 | | Cross-validation 3 | | Cross-validation 4 | | Cross-validation 5 | |
| --- | --- | --- | --- | --- | --- | --- | --- | --- | --- | --- |
| Training set | 18942/46584 | 0.4 | 17803/42221 | 0.4 | 17908/42385 | 0.4 | 19013/47664 | 0.4 | 18556/46133 | 0.4 |
| Validation set | 6326/15517 | 0.4 | 5853/14156 | 0.4 | 6027/14071 | 0.4 | 6323/15903 | 0.4 | 6175/15388 | 0.4 |
| Testing set | 5897/13443 | 0.4 | 7509/19167 | 0.4 | 7230/19088 | 0.4 | 5829/11977 | 0.5 | 6434/14023 | 0.5 |

Table S9 Hyperparameter in Compared Models

| Model | Hyperparameter | | Values Range | | Values |
| --- | --- | --- | --- | --- | --- |
| DeepSynergy | Drug encoder channels | | [32,64,96,128] | | 128 |
|  | Context encoder channels | | [32,64,96,128] | | 128 |
|  | Hidden layer channels | | [32,64,96,128] | | {128,128,128} |
|  | dropout rate | | [0,0.5] | | 0.41 |
|  | learning rate | | [10^-5^,10^-3^] | | 5.15e-05 |
| MatchMaker | Drug encoder channels | [32,64,96,128] | | {96,96} | |
|  | Hidden layer channels | [32,64,96,128] | | {96,96} | |
|  | drop rate | [0,0.5] | | 0.078 | |
|  | learning rate | [10^-5^,10^-3^] | | 0.00031 | |
| MRGNN | Drug encoder channels | [32,64,96,128] | | 96 | |
|  | Hidden layer channels | [32,64,96,128] | | 96 | |
|  | Drug encoder layers | [2,4,8,16 | | 4 | |
|  | learning rate | [10^-5^,10^-3^] | | 0.00058 | |
| GCNBMP | Drug encoder channels | [32,64,96,128] | | 64 | |
|  | Hidden layer channels | [1,2,3,4,5] | | 64 | |
|  | learning rate | [10^-5^,10^-3^] | | 0.00079 | |
| EPGCNDS | Drug encoder channels | [32,64,96,128] | | 128 | |
|  | Hidden layer channels | [32,64,96,128] | | 96 | |
|  | learning rate | [10^-5^,10^-3^] | | 0.00099 | |
| DeepDDS | Context encoder channels | [32,1024],step=256,  [32,512],step=128,  [32,128],step=32 | | [800,160,64] | |
|  | Hidden layer channels | [32,64,96,128],  [32,64,96,128] | | [128,96] | |
|  | learning rate | [10^-5^,10^-3^] | | 0.00063 | |
|  | dropout | [0,0.5] | | 0.25 | |

Table S10 Compared methods With r-drop and Without r-drop in DrugcombDB

| Model | ACC | BACC | Prec | Rec | F1 | ROC-AUC | MCC | KAPPA | AP |
| --- | --- | --- | --- | --- | --- | --- | --- | --- | --- |
| DFFNDDS | 0.871  (0.002) | 0.834  (0.002) | 0.801  (0.008) | 0.746  (0.006) | 0.773  (0.003) | 0.921  (0.003) | 0.684  (0.004) | 0.683  (0.004) | 0.859  (0.005) |
| DFFNDDS(w.o.rdrop) | 0.866  (0.002) | 0.832  (0.005) | 0.785  (0.010) | 0.750  (0.017) | 0.767  (0.006) | 0.919  (0.003) | 0.673  (0.007) | 0.673  (0.007) | 0.853  (0.007) |
| DeepSynergy | 0.821  (0.005) | 0.750  (0.009) | 0.758  (0.007) | 0.576  (0.013) | 0.654  (0.017) | 0.750  (0.009) | 0.546  (0.005) | 0.537  (0.006) | 0.561  (0.002) |
| DeepSynergy(w.o.rdrop) | 0.827  (0.003) | 0.762  (0.005) | 0.760  (0.010) | 0.604 (0.011) | 0.673 (0.009) | 0.860  (0.002) | 0.565 (0.010) | 0.558  (0.010) | 0.773  (0.008) |
| MatchMaker | \| 0.820  (0.006) \| \| --- \| | 0.738  (0.009) | 0.777  (0.013) | 0.542  (0.019) | 0.638  (0.015) | 0.848  (0.008) | 0.538 (0.016) | 0.523 (0.017) | 0.754 (0.014) |
| MatchMaker(w.o.rdrop) | 0.762  (0.005) | 0.638  (0.009) | 0.698  (0.006) | 0.339  (0.003) | 0.454  (0.002) | 0.746  (0.007) | 0.360  (0.012) | 0.325  (0.005) | 0.568  (0.002) |
| MRGNN | \| 0.811  (0.003) \| \| --- \| | 0.745  (0.002) | 0.721  (0.008) | 0.585  (0.005) | 0.646  (0.003) | 0.745  (0.002) | 0.524  (0.006) | 0.519  (0.005) | 0.544  (0.003) |
| MRGNN(w.o.rdrop) | 0.810  (0.003) | 0.737  (0.006) | 0.730  (0.010) | 0.560 (0.017) | 0.634  (0.008) | 0.845  (0.001) | 0.517 (0.007) | 0.508  (0.008) | 0.734  (0.004) |
| GCNBMP | 0.776  (0.003) | 0.700  (0.014) | 0.652  (0.018) | 0.516  (0.014) | 0.575  (0.002) | 0.700  (0.004) | 0.432  (0.014) | 0.426 (0.012) | 0.479  (0.014) |
| GCNBMP(w.o.rdrop) | 0.802  (0.002) | 0.717  (0.006) | 0.737  (0.014) | 0.511  (0.018) | 0.603  (0.010) | 0.828  (0.005) | 0.492(0.006) | 0.477  (0.007) | 0.710  (0.007) |
| EPGCNDS | \| 0.751  (0.004) \|  \|  \|  \|  \| \| --- \| --- \| --- \| --- \| --- \| | 0.645  (0.011) | 0.624  (0.017) | 0.388  (0.040) | 0.477  (0.024) | 0.645  (0.011) | 0.342  (0.009) | 0.325  (0.015) | 0.421 (0.008) |
| EPGCNDS(w.o.rdrop) | 0.754  (0.003) ) | 0.630  (0.013) | 0.672  (0.028) | 0.328 (0.039) | 0.438  (0.031) | 0.740  (0.002) | 0.338  (0.012) | 0.305  (0.022) | 0.583  (0.007) |
| DeepDDS | \| 0.831  (0.002) \| \| --- \| | 0.748  (0.005) | 0.820  (0.010) | 0.546  (0.012) | 0.655 (0.007) | 0.873  (0.001) | 0.569  (0.006) | 0 .549  (0.008) | 0.779  (0.006) |
| DeepDDS(w.o.rdrop) | 0.852  (0.002) | 0.784  (0.004) | 0.838  (0.010) | 0.617  (0.011) | 0.711  (0.006) | 0.898 (0.002) | 0.628 (0.005) | 0.615  (0.006) | 0.826  (0.004) |
